# Magnitude and Associated Factors of Surgical Site Infection Following Cesarean Section: A Cross‐Sectional Study in Southern Ethiopia

**DOI:** 10.1002/hsr2.73188

**Published:** 2026-09-03

**Authors:** Kidane Tsegaw, Tesfaw Alemu Legas, Amene Abebe Kerbo, Belachew Shote Garbo, Samuel Minalkew, Tigist Nega Alemu, Wondimagegn Genaneh Shiferaw

**Affiliations:** ^1^ School of Medicine Wolaita Sodo University Sodo City Ethiopia; ^2^ Department of Midwifery Wolaita Sodo University Sodo City Ethiopia; ^3^ School of Public Health Wolaita Sodo University Sodo City Ethiopia; ^4^ Department of Nursing Wolaita Sodo University Sodo City Ethiopia; ^5^ Department of Emergency and Critical Care Nursing Wolaita Sodo University Sodo City Ethiopia

**Keywords:** associated factors, cesarean delivery, post‐cesarean section, risk factors, Southern Ethiopia, surgical site infection

## Abstract

**Background and Aim:**

Surgical site infections (SSIs) occur at the incision site or in deeper tissues within 30 days of surgery and represent a significant public health concern, contributing substantially to maternal morbidity and mortality. Evidence on the magnitude and associated factors of SSIs among women undergoing cesarean delivery in this setting remains limited, and several potentially influential variables have not yet been explored. Therefore this study aimed to determine the prevalence and associated factors of post‐cesarean section surgical site infection in Southern Ethiopia.

**Methods:**

An institution‐based cross‐sectional study was conducted among mothers who underwent cesarean section at WSUCSH from March 1 to September 1, 2024. Surgical site infection (SSI) was defined as an infection developing at the cesarean delivery operative site within 30 days of surgery, diagnosed by the attending clinician. A systematic random sampling technique was used to select 275 patient records from all cesarean delivery cases during the study period. Data were entered into Epi‐Data and analyzed using SPSS version 25. Binary logistic regression was performed to identify factors associated with the outcome variable. Adjusted odds ratios (AORs) with 95% confidence intervals (CIs) were calculated to measure the strength of associations, with statistical significance set at *p* < 0.05.

**Results:**

The overall post‐cesarean SSI rate was 8.8% (95% CI: 5.0–12.0). Prolonged operative duration (AOR = 3.27, 95% CI: 1.11–9.61), prolonged rupture of membranes prior to surgery (AOR = 3.53, 95% CI: 1.23–10.14), presence of chorioamnionitis (AOR = 4.36, 95% CI: 1.06–17.86), inadequate vaginal antiseptic preparation (AOR = 4.32, 95% CI: 1.13–16.48), excessive intraoperative blood loss (AOR = 3.73, 95% CI: 1.34–10.39), and a postoperative hematocrit below 30% (AOR = 4.34, 95% CI: 1.47–12.85) were identified as independent risk factors for SSI.

**Conclusions:**

Post‐cesarean SSI remains a notable concern in this setting. Minimizing operative time, ensuring adequate vaginal antiseptic preparation, promptly managing prolonged membrane rupture, early treatment of intra‐partum infections, and correction of maternal anemia are critical strategies for reducing post‐cesarean SSI.

**Abbreviations**
CS: cesarean section
DM: diabetes mellitus
HAI: hospital acquired infections
HTN: hypertension
IRB: Institutional Review Board
OB/GYN: obstetrics and gynecology
ROM: rupture of membrane
SSI: surgical site infection
WSUCSH: Wolaita Sodo University Comprehensive Specialized Hospital

## 1 Introduction

Surgical site infections are infections that occur at the incision site, underlying tissues, or organs within 30 days of a surgical procedure or within 1 year if an implant is involved [1]. SSIs are increasingly difficult to treat due to rising antimicrobial resistance and patient comorbidities, and their prevention has become more urgent as surgical volumes grow worldwide [2]. Cesarean delivery (CD) is particularly prone to SSIs, which typically develop within 30 days postoperatively and are a major cause of maternal morbidity and mortality, presenting as wound discharge, dehiscence, or necrotizing fasciitis [3, 4]. Established risk factors include prolonged labor, duration of membrane rupture, frequency of vaginal examinations, and low hemoglobin [3, 5, 6].

Globally, SSIs affect 2%–5% of surgical patients and are among the most common healthcare‐associated infections (HAIs), with LMICs bearing a disproportionate share, affecting up to one‐third of surgical patients [6]. Nearly half of SSIs are considered preventable through evidence‐based strategies, and public reporting of outcomes and quality‐improvement initiatives are now emphasized to address this [1, 2]. The WHO's global guidelines for safe surgery recommend prophylactic antibiotics within 60 min of incision, sterility monitoring during instrument sterilization, pre‐surgical skin antisepsis, and a surgical safety checklist as essential measures to reduce SSIs [2, 7].

Surgical site infections (SSIs) remain a significant complication following cesarean delivery (CD) worldwide, with an estimated global incidence of 5.63% [8]. The prevalence varies widely between countries, ranging from 0.15% in China [9], to 12.6% in Nepal [10]. In India, the rate is reported at 8.02% [11], while in the United Arab Emirates, it is 1.4% [12]. In low‐ and middle‐income countries (LMICs), a WHO report identified an overall prevalence of 11.2 [13]. In Africa, the pooled prevalence of SSIs following cesarean delivery is 10.21% [14], with Sub‐Saharan Africa reporting a slightly lower rate of 7.3% [15]. In Ethiopia, studies show an SSI prevalence ranging from 8.81% to 12.32% after C/S delivery [16, 17]. The cross‐country range in reported SSI prevalence likely reflects differences in SSI case definitions, surveillance intensity, length of post‐discharge follow‐up, and population characteristics, and that these figures should not be compared directly.

As global surgical volumes rise, cesarean section remains one of the most common procedures [1, 18], and SSIs affect nearly one‐third of surgical patients, posing a substantial financial and human burden [5], with mortality estimated at 1.33% [19]. The economic burden is considerable: in USA, SSIs are associated with a 9.7‐day extended hospital stay and an added $20,842 per admission, and nationally account for over 400,000 extra hospital days, more than $900 million in costs, and roughly $700 million in SSI‐related readmission costs [1]; comparable cost data specific to LMIC or African settings are not available.

In low‐ and middle‐income countries, the incidence of SSIs is significantly higher than in high‐income countries, largely due to inadequate infection prevention practices. According to the WHO, healthcare‐associated infections are two to three times more common in LMICs compared to high‐income nations. It is estimated that nearly half of SSIs could be prevented through evidence‐based interventions [1, 13]. In Ethiopia, common risk factors for post‐cesarean SSI include prolonged labor, premature rupture of membranes, anemia, chorioamnionitis, and general anesthesia [19]. These infections lead to increased maternal morbidity and mortality, prolonged hospital stays, and higher healthcare costs. Limited healthcare resources, poor infrastructure, and inadequate infection control practices further complicate the problem. Addressing SSIs is vital to improving maternal health outcomes in the country [5, 14, 17].

Despite this evidence base, most Ethiopian studies have focused on a narrow set of conventional risk factors such as prolonged labor and anemia, while variables including preoperative skin and vaginal antiseptic preparation, duration of prophylactic antibiotic use, frequency of vaginal examinations, and postoperative length of stay remain largely unexplored; evidence from the Wolaita Zone specifically is virtually absent. This study was therefore conducted to determine the magnitude and independent predictors of post‐cesarean delivery SSI at WSUCSH, with particular attention to these understudied variables, to generate actionable, context‐specific evidence for maternal surgical care in this setting.

## 2 Materials and Methods

### 2.1 Study Area and Period

The study was conducted at Wolaita Sodo University Comprehensive Specialized Hospital (WSUCSH) in Wolaita Sodo Town, Southern Ethiopia. The hospital is located 332 kilometers south of Addis Ababa, the capital city of Ethiopia. WSUCSH serves as a teaching hospital for both undergraduate and postgraduate programs. It provides healthcare services to a catchment population exceeding 5 million and has a capacity of 350 inpatient beds [20, 21]. The hospital is equipped with three major operating rooms and an intensive care unit comprising eight beds shared among various departments. The study was carried out over a 6‐month period, from March 1 to September 1, 2024.

### 2.2 Study Design

An institution‐based cross‐sectional study was conducted.

### 2.3 Population

#### 2.3.1 Source Population

The source population comprised all mothers who underwent cesarean section at Wolaita Sodo University Comprehensive Specialized Hospital.

#### 2.3.2 Study Population

The study population included all mothers who underwent CS at WSUCSH during the study period.

### 2.4 Inclusion and Exclusion Criteria

#### 2.4.1 Inclusion Criteria

Medical records of mothers who underwent CS within the specified study period were included.

#### 2.4.2 Exclusion Criteria

✓ Medical records with incomplete or missing data on key outcome variables were excluded.
✓ Mothers who underwent CS at another institution.

### 2.5 Sample Size

The sample size for the first objective calculated using single population proportion formula considering P of 11% taken from previous study done in Hawasa, Ethiopia [22], with the assumption of 95% CI, margin of error 4% (d = 0.04). Then adding 10% non‐response rate.

n = Za^2^P(1‐P)/d^2^_where_;

*n* = Total sample size, z = desired level of confidence interval 95% (1.96), p = magnitude of Surgical Site infection after C/S was 11%, d = margin of error which correspond to the level of precision of results desired = 4% and non‐response rate = 10% then *n* = (1.96)^2^ (0.11) (0.89)/ (0.04)^2^ = 235, then considering non‐response rate of 10%, *n* = 235 + 24 = 259.

The sample size for the second objective calculated with Open Epi info using assumption of power 80%, 95% CI and exposed/ unexposed ratio of 1:1. (*see* Table 1 *for more information*).

**Table 1:** Sample size determination for the second objective for factors associated with the post CS surgical site infection in Wolaita Sodo University Comprehensive Specialized Hospital, Wolaita, Southern Ethiopia, 2024.

Variables	% of outcome unexposed	% of exposed	APOR	Sample size with 10% non‐response	Reference
Chorioamnionitis	66	8	6.1	26	[3]
Preoperative HCT	11	33	0.2	128	[23]
Duration of labor	25	6	0.5	114	[23]

In generally, the largest sample size was considered to minimize the sampling error. As the result, the first objective sample value is used to this study, that is, (*n* = 259).

### 2.6 Sampling Procedure

In this study, a systematic random sampling technique was utilized to select participants from a total population of 1081 women who underwent cesarean section at Wolaita Sodo University Comprehensive Specialized Hospital between March 1 and September 1, 2024. The operation theater logbook served as the sampling frame, listing all eligible patient records with unique sequential registration numbers. A sampling interval of four was calculated by dividing the total population by the desired sample size (k = 1081 ÷ 259 ≈ 4). The first participant was selected randomly from the first interval using a lottery method, and thereafter every fourth record was systematically selected until the required sample participants were achieved. The calculated minimum sample size was 259; we drew 275 records to build in a margin for anticipated incomplete records, 25 of which (9.1%) were subsequently excluded, yielding a final analytic sample of 250. Prior to selection, the eligibility of records was confirmed to ensure that only complete and accurate data were included in the final analysis.

### 2.7 Study Variable

#### 2.7.1 Dependent

Post C/S Surgical site infection.

#### 2.7.2 Independent

**Socio demographic factors**‐sex and residency.

**Preoperative factors**‐membrane status, duration of ROM, chorioamnionitis, duration of labor, co‐morbidity, vaginal preparation, number of vaginal examinations, preoperative hemoglobin.

**Surgery related factors**‐type of operation, duration of surgery, prophylaxis antibiotic, type of skin preparation, anesthesia.

**Post operative factors**‐duration of IV antibiotics, duration of hospital stays.

### 2.8 Operational Definition

**Surgical site infections** are infections that occur at the incision site and/or deeper underlying tissue spaces and organs within 30 days of a surgical procedure (or within 1 year of implant) [24].

**Post cesarean section surgical site infection:** An infection which is developed after cesarean delivery on the operational site which is diagnosed by clinician [2].

**Prolonged hospital stay;** defined as hospital stay lasting more than 7 days [25].

**Cesarean section**‐ defines the birth of a fetus by laparotomy and then hysterotomy after fetal viability [26].

**Antibiotic prophylaxis:** The administration of antibiotics before surgery to prevent infections [27].

### 2.9 Data Collection

Data collection was conducted from March 1 and September 1, 2024, using a structured checklist prepared in English. The checklist was developed by the principal investigator based on relevant literature and pretested to ensure clarity and comprehensiveness. Four midwives were recruited as data collectors. Prior to data collection, they received a 1‐day training session that covered the study objectives, the importance of collecting accurate and reliable data, and ethical considerations during chart review. The training also included a detailed walkthrough of the checklist, with explanations and clarifications provided for each question to ensure consistency and accuracy.

The data collection focused on key variables, including socio‐demographic characteristics, preoperative risk factors, surgery‐related factors, and postoperative risk factors. These were extracted from the medical records of eligible patients using the pretested data collection tool.

The principal investigator provided continuous supervision throughout the data collection process to ensure adherence to protocols and address any issues that arose promptly. Regular feedback sessions were conducted with data collectors to resolve challenges and maintain data quality.

### 2.10 Data Quality Assurance

To ensure data quality, all collected data were checked for completeness by the data collectors immediately after extraction and cross‐verified the data for accuracy and consistency by Investigator.

A pretest was conducted on 5% of the total sample size at Wachamo Comprehensive Specialized Hospital prior to the actual data collection. This pretest allowed for assessing the relevance, clarity, and contextual appropriateness of the data collection tool. Based on the pretest results, necessary modifications and corrections were made to enhance the tool's reliability and validity.

### 2.11 Data Management and Analysis

The collected data were entered into Epi‐Data version 3.1 and then exported to SPSS version 25 for statistical analysis. Descriptive statistics, including frequencies and percentages, were used to summarize categorical variables. The results were presented in tables, graphs, and charts for clarity and easy interpretation. Multicollinearity among predictor variables was assessed using correlation coefficients to ensure that the variables included in the regression models were independent.

To identify factors associated with SSI, a two‐stage logistic regression analysis was performed. Initially, bivariable logistic regression was conducted to examine the relationship between each independent variable and the outcome variable. Variables associated with the outcome at *p* ≤ 0.25 in bivariable analysis were considered for inclusion in the multivariable model, following a data‐driven (exploratory) variable‐selection approach rather than a pre‐specified hypothesis‐driven approach. Subsequently, a multivariable analysis was conducted to control for confounding factors and identify independent predictors of SSI. The results were expressed as adjusted odds ratios with 95% confidence intervals, and a *p*‐value < 0.05 was considered statistically significant for SSI after c/s. All statistical tests were two‐sided.

### 2.12 Ethical Consideration

Ethical approval for this study was obtained from the Ethical Review Committee of Wolaita Sodo University, College of Health Sciences and Medicine, with approval number CHSM/ERC/02/15. Following the approval, an official permission letter was obtained from the administration of Wolaita Sodo University Comprehensive Specialized Hospital to carry out the research. The hospital administration was fully informed about the study's objectives, methodologies, and data collection procedures to ensure transparency and cooperation. Informed consent was waived for this study as it involved a retrospective review of existing patient charts and records, with no direct patient contact or intervention.

In order to protect patient confidentiality, all data were anonymized, and patient names were excluded from the data collection process. Stringent measures were taken to ensure the confidentiality of all collected data, which were securely stored throughout the study period. The results of this study will be used solely for research purposes.

## 3 Results

### 3.1 Socio‐Demographic and Clinical Characteristics of the Study Participants

Among the 250 women included in the analysis, the mean age was 25 ± 5 years (range: 18–36), with 212 (84.8%) aged 20–34 years. More than half, 131 (52.4%), resided in urban areas. Comorbidities were reported in 38 (15.2%) participants, most commonly hypertension (4.8%), anemia (3.2%), and obesity (2.4%), while 212 (84.8%) had no comorbid conditions. (*see* Table 2 *for more information*).

**Table 2:** The distribution of socio‐demographic and clinical characteristics of women underwent c/s delivery at Wolaita Sodo University Compressive Specialized Hospital, Wolaita, Southern Ethiopia, 2024.

Characteristics	Categories	Frequency (*n* = 250)	Percent (%)
Age	≤ 19 Years	11	4.4
Presence of Comorbidity disease	20–34 Years	212	84.8
	≥ 35 Years	27	10.8
Residence	Urban	131	52.4
	Rural	119	47.6
Hypertension	Yes	12	4.8
	No	238	95.2
Anemia	Yes	8	3.2
	No	242	96.8
Obesity	Yes	6	2.4
	No	244	97.6
DM	Yes	4	1.6
	No	246	98.4
HIV/AIDS	Yes	3	1.2
	No	247	98.8
Others	Yes	5	2.0
	No	245	98.0

### 3.2 Obstetrics Characteristics of Participants

Among the 250 participants (97.6%) had antenatal care (ANC) follow‐up, with 84.4% attending four or more visits, while 13.2% attended 1–3 visits. Regarding the place of ANC visits, 51.2% attended health centers, 42.8% visited hospitals, and a smaller proportion attended private clinics (2.8%) or other facilities (0.8%).

Before undergoing the operation, 89.2% of mothers had been in labor, and 72.8% had undergone more than five vaginal examinations, while 24.0% had five or fewer examinations. The membranes were intact in 57.2% of cases, while 42.8% had ruptured membranes. Chorioamnionitis was reported in 7.6% of participants, and vaginal preparation was performed in 34.4%, with the remaining 65.6% not receiving vaginal preparation. (*See* Table 3 *for more information*).

**Table 3:** Obstetrics characteristics of the women who underwent c/s in Wolaita Sodo University Compressive Specialized Hospital, Wolaita, Southern Ethiopia, 2024.

Characteristics	Categories	Frequency (*n* = 250)	Percent (%)
ANC follow‐up	Yes	244	97.6
	No	6	2.4
Number of ANC visits	1–3x	33	13.2
	≥ 4x	211	84.4
Place of ANC visits	Hospital	107	42.8
	Health center	128	51.2
	Private clinic	7	2.8
	Others	2	0.8
Mother has been in labor before operation	Yes	223	89.2
	No	27	10.8
Vaginal examination done	No exam	8	3.2
	Yes (≤ 5x)	60	24.0
	Yes (> 5x)	182	72.8
Membrane status	Intact	143	57.2
	Ruptured	107	42.8
Chorioamnionitis	Yes	19	7.6
	No	231	92.4
Vaginal preparation	Yes	86	34.4
	No	164	65.6

### 3.3 Operation‐Related Characteristics of Participants

Among the 250 participants, the majority (89.2%) underwent emergency cesarean sections (C/S), while 10.8% had elective procedures. Spinal anesthesia was predominantly used (96.4%), with general anesthesia administered in only 3.6% of cases. Most skin incisions were transverse (94.0%), and 6.0% were vertical.

Iodine‐alcohol was the primary antiseptic used before surgery in 97.6% of cases. Junior surgeons (R1/R2) performed 32.0% of the procedures, while senior surgeons (R3/R4) conducted the remaining 68.0%. A majority of surgeries (73.2%) lasted less than 60 min, with 26.8% extending beyond an hour. Blood loss during surgery was estimated at less than 500 mL in 66.4% of cases, while 33.6% experienced blood loss exceeding 500 ml.

Postoperatively, 67.2% of participants maintained a hematocrit level of 30% or higher, and 93.6% received antibiotic prophylaxis. Intravenous antibiotics were administered to 60.0% of participants, while 40.0% received both IV and oral antibiotics. The intravenous antibiotic course was limited to three doses in 56.4% of cases, while 12.0% received a combined IV and oral antibiotic regimen lasting 7 days or more. Most patients (96.0%) were discharged within 7 days, with 4.0% staying longer. (*See* Table 4 *for more information*).

**Table 4:** Operation‐related characteristics of women who underwent C/S in Wolaita Sodo University Compressive Specialized Hospital, Wolaita, Southern Ethiopia, 2024.

Characteristics	Categories	Frequency (*n* = 250)	Percent (%)
Indication for C/S	NRFHR	90	36.0
	LFSOL+GIIIMSAF	60	24.0
	1C/S Scar+x‐Factor	25	10.0
	Malpresentation/Malposition	20	8.0
	2C/S Scar	16	6.4
	CPD	12	4.8
	Others	27	10.8
Type of C/D	Emergency	223	89.2
	Elective	27	10.8
Type of anesthesia used	General	9	3.6
	Spinal	241	96.4
Type of skin incision	Vertical	15	6.0
	Transverse	235	94.0
Types of anti‐septic used before operation	Iodine	6	2.4
	Iodine‐Alcohol	244	97.6
Level of surgeon	Junior(R1/R2)	80	32.0
	Senior(R3/R4)	170	68.0
Time taken for surgery	< 60 min	183	73.2
	≥ 60 min	67	26.8
Estimated blood loss	< 500 ML	166	66.4
	≥ 500 ML	84	33.6
Post‐operative HCT	< 30%	82	32.8
	≥ 30%	168	67.2
ABS prophylaxis given	Yes	234	93.6
	No	16	6.4
Post‐operative therapeutic ABS	IV only	150	60.0
	Both IV and oral	100	40.0
Duration of IV ABS	≤ 3 dose	141	56.4
	> dose	9	3.6
Duration of both ABS (IV& Oral)	< 7 days	70	28.0
	≥ 7 days	30	12.0
Hospital stay duration after C/S	≤ 7 days	240	96.0
	> 7days	10	4.0

### 3.4 Indication for C/S of the Study Participants

**Figure 1:**
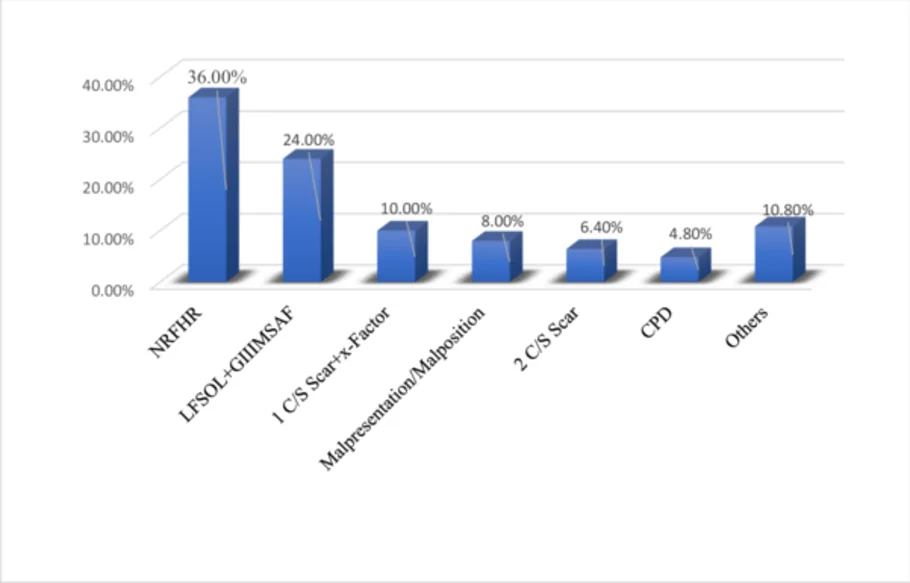
Distribution of indication for C/S delivery that underwent surgery at Wolaita Sodo Compressive Specialized Hospital, Wolaita Zone, South Ethiopia, 2024. C/S, cesarean section; CPD, cephalopelvic disproportion; LFSOL+GIIIMSAF, latent first stage of labor with grade III meconium‐stained amniotic fluid; NRFHR, non‐reassuring fetal heart rate.

Among the 250 participants, the most common indications for cesarean section were non‐reassuring fetal heart rate (36.0%), followed by latent first stage of labor with grade III meconium‐stained amniotic fluid (24.0%), and one previous C/S scar with an additional risk factor (10.0%) (*see* Figure 1 *for more information*).

### 3.5 Magnitude of Surgical Site Infection

**Figure 2:**
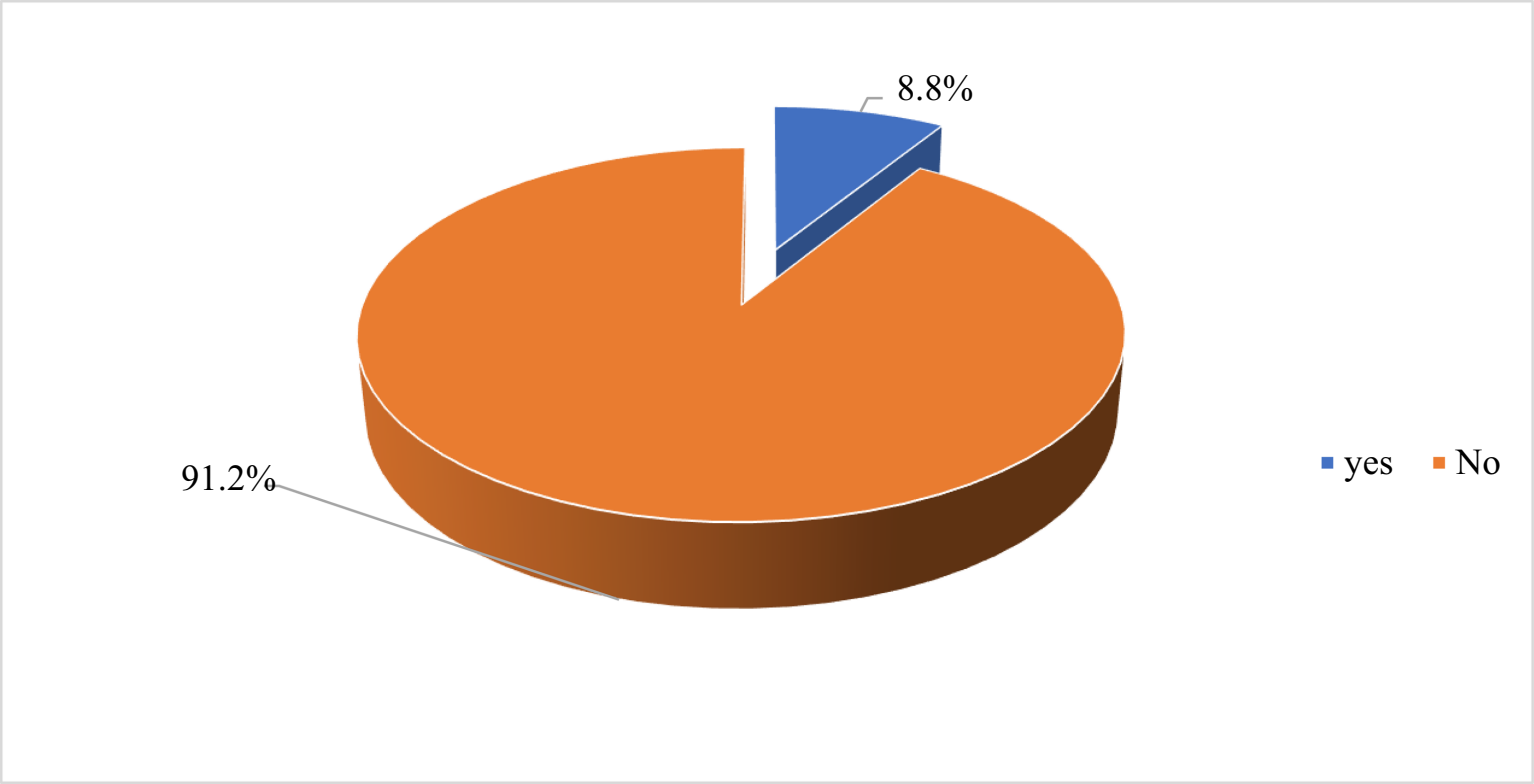
Magnitude Of post C/S infection among women who underwent C/S infection in Wolaita Sodo Compressive Specialized Hospital, Wolaita Zone, South Ethiopia, 2024. Overall SSI prevalence was 8.8% (22/250); 18 cases were identified before discharge and 4 after discharge.

The magnitude of surgical site infection following cesarean section was found to be 22 cases, overall SSI prevalence (8.8%, 95% CI 5.0%–12.0%). Of these, 18 cases were identified before discharge, while four cases were detected after discharge. (*See* Figure 2 *for more information*).

### 3.6 Risk Factors of Surgical Site Infection

Bivariable and multivariable logistic regression analyzes were conducted to identify risk factors for surgical site infection. In the bivariable analysis, variables with a *p*‐value < 0.25 were included in the multivariable model. From the total independent variables, eight variables were selected for multivariable analysis: duration of the operation, type of anesthesia, vaginal preparation, membrane status, chorioamnionitis, type of skin incision, post‐operative hematocrit, and estimated blood loss.

Multivariable logistic regression analysis identified six significant predictors of SSI following cesarean section at a *p*‐value < 0.05. The absence of vaginal preparation was associated with an increased risk of developing SSI. Prolonged operation duration significantly elevated the likelihood of infection. Ruptured membranes were also identified as a key risk factor. Additionally, the presence of chorioamnionitis notably increased the risk of SSI. Low post‐operative hematocrit levels (< 30%) were linked to higher susceptibility to infection, prolonged surgical duration (> 60 min), and substantial blood loss during surgery (≥ 500 mL) further contributed to the risk of SSI.

Several factors were significantly associated with an increased risk of post‐cesarean surgical site infection (SSI). Women who underwent prolonged surgical duration were three times more likely to develop SSI compared to those with shorter durations of surgery ([AOR = 3.265, 95% CI: 1.109–9.608, *p* = 0.032]). Similarly, those with ruptured membranes (ROM) had 3.5 times higher odds of developing SSI compared to women with intact membranes ([AOR = 3.532, 95% CI: 1.230–10.144, *p* = 0.019]). The presence of chorioamnionitis further heightened the risk, making women four times more likely to develop SSI ([AOR = 4.358, 95% CI: 1.063–17.864, *p* = 0.041]).

Lack of vaginal preparation before cesarean surgery was another critical factor, with affected women being four times more likely to develop SSI compared to those who underwent proper preparation ([AOR = 4.320, 95% CI: 1.132–16.481, *p* = 0.032]). Excessive intraoperative blood loss (≥ 500 mL) also increased the likelihood of SSI by threefold ([AOR = 3.730, 95% CI: 1.339–10.392, *p* = 0.012]). Finally, postoperative hematocrit levels below 30% were associated with a fourfold higher risk of developing SSI compared to levels exceeding 30% ([AOR = 4.338, 95% CI: 1.465–12.848, *p* = 0.008]). (*See* Table 5 *for more information*).

**Table 5:** Bivariate and multivariable logistic regression analysis of post c/s infection among women underwent C/S in Wolaita Sodo University Compressive Specialized Hospital, Wolaita, Southern Ethiopia, 2024.

Variables		Post C/S SSI		COR (95% CI)	AOR (95% CI)	*p* value
		Yes (%)	No (%)			
Duration of the operation	< 60 min	13 (59.1)	170 (74.6)	1	1	
	≥ 60 min	9 (40.9)	58 (25.4)	2.029 (0.824, 4.994)	3.265 (1.109, 9.608)	0.03
Types of anesthesia	General	3 (13.6)	12 (2.6)	1	1	
	Spinal	19 (86.4)	222 (97.4)	5.842 (1.353, 25.230)	5.230 (0.732, 37.348)	0.02
Vaginal preparation	Yes	4 (18.2%)	82 (36.0)	1	1	
	No	18 (81.8%)	146 (64.0%)	2.527 (0.827, 7.720)	4.320 (1.132, 16.481)	0.03
Membrane status	Intact	8 (36.4)	135 (59.2)	1	1	
	Ruptured	14 (63.6)	93 (40.8)	2.540 (1.025, 6.298)	3.532 (1.230, 10.144)	0.02
Chorioamnionitis	Yes	4 (18.2)	15 (6.6)	3.156 (0.947, 10.511)	4.358 (1.063, 17.864)	0.04
	No	18 (81.8)	213 (93.4)	1	1	
Types of skin incisions	Vertical	3 (13.6)	12 (5.3)	2.842 (0.737, 10.955)	3.747 (0.688, 20.406)	0.13
	Transverse	19 (86.4)	216 (94.7)	1	1	
Post‐operative HCT	< 30%	15 (68.2)	67 (29.4)	5.149 (2.009, 13.199)	4.338 (1.465, 12.848)	0.01
	≥ 30%	7 (31.8)	161 (70.6)	1	1	
Estimated blood loss	< 500 ml	8 (36.4)	158 (69.3)	1	1	
	≥ 500 ml	14 (63.6)	70 (30.7)	3.950 (1.585, 9.844)	3.730 (1.339, 10.392)	0.01

## 4 Discussion

This study reveals a concerning prevalence of 8.8% for surgical site infections among mothers who underwent cesarean sections at Wolaita Sodo University Comprehensive Specialized Hospital, with a confidence interval of 95% (5.0%–12.0%). This rate requires urgent need to address SSI as a critical public health issue, particularly in low‐income settings like Ethiopia, where maternal morbidity and mortality rates are alarmingly high. The economic burden associated with prolonged hospitalization and increased workload for healthcare providers further complicates the challenges faced by families and the healthcare system. Therefore, understanding the magnitude and risk factors of post‐cesarean section SSI is essential for developing effective prevention strategies. By identifying these determinants, this study aims to inform healthcare policies and practices that can mitigate risks and enhance patient care, ultimately contributing to improved maternal health outcomes in our hospitals.

The prevalence of SSIs in this study (8.8%) aligns with findings from several studies conducted in Ethiopia, including in Assela (9.4%) [28], in Zewditu Memorial Hospital (8.4%) [29], Debre Tabor (8%) [30], Hawassa(11%) [22], FelegeHiwot Referral Hospital (7.8%) [31], in Mekele (11.7%) [32], and Lemlem Karl Hospital (6.8%) [33]. Similarly, it is comparable with studies from other countries, such as Eritrea (6.8%) [27], Tanzania (10.9%) [34], Nigeria (9.1%) [35], Rwanda (10.9%) [36], Kosovo (9.85%) [37] and Sierra Leone (10.9%) [38].

On the other hand, the prevalence reported here is slightly lower than findings from Malaysia (18.8%) [10] and Uganda (15.5%) [39]. This difference might be due to variations in data collection methods, as this study relied on secondary data, potentially underestimating SSI prevalence due to unrecorded outcomes, whereas those studies used direct patient follow‐ups.

Conversely, the prevalence in this study was higher than results reported in Egypt (2.87%) [39], Kuwait (2.1%) [40], Gujarat (4.25%) [9], Peru (2.4%) [18] and Brazil (1.44%) [41]. The observed discrepancies could be attributed to differences in socio‐economic conditions, healthcare infrastructure, infection prevention measures, and health policies across regions.

The study identified several key associated factors significantly associated with the occurrence of surgical site infection (SSI) following cesarean delivery. These include the absence of vaginal preparation, ruptured membranes, presence of chorioamnionitis, and low postoperative hematocrit levels (< 30%). Additionally, prolonged surgical duration (> 60 min) and considerable intraoperative blood loss (≥ 500 mL) were found to markedly increase the risk of SSI.

Rupture of membranes before cesarean section was found to be an independent risk factor for SSI, with women experiencing this condition being 3.5 times more likely to develop SSI. This finding was Supported study conducted in different part of Ethiopia [5, 22, 28, 33, 42]. A possible explanation is that ruptured membranes increase the risk of ascending infections by providing a direct pathway for pathogens to the uterine cavity and surgical site. Additionally, prolonged rupture of membranes before surgery can lead to a higher bacterial load and inflammation, which compromise tissue healing and elevate the risk of post‐operative infection.

Women with a history of chorioamnionitis during the current birth were 4.4 times more likely to develop post‐cesarean surgical site infections (SSI) compared to those without chorioamnionitis (AOR = 4.358, 95% CI: 1.063–17.864). This finding is supported with studies conducted in [17, 23, 28, 32, 43]. The possible explanation might be chorioamnionitis, an intra‐amniotic infection, likely increases the risk of SSI through several mechanisms. The infection elevates systemic inflammation and bacterial load, both of which can persist post‐operatively, heightening the risk of wound contamination. Additionally, prolonged rupture of membranes and frequent vaginal examinations, often associated with chorioamnionitis, can introduce pathogens that exacerbate the likelihood of infection and also compromised immune responses and the surgical complexity involved in managing infected or inflamed tissues further contribute to delayed wound healing and increased infection risk.

Women who experienced excessive intraoperative blood loss (≥ 500 ml) had 3.7 times higher odds of developing post‐cesarean section SSI compared to those with lower blood loss. This finding is supported by findings from other study [44]. The reason might be excessive blood loss during surgery can lead to hypovolemia and reduced tissue oxygenation, impairing the healing process and increasing susceptibility to infections. Moreover, significant blood loss often necessitates prolonged surgical procedures and the use of invasive devices, which may introduce pathogens into the surgical site. The immunosuppressive effects of acute blood loss, potential bacterial contamination during blood transfusions, and compromised hemostasis further delay wound healing and create conditions favorable for bacterial proliferation, increasing the likelihood of SSI.

In this study, a cesarean section lasting longer than 60 min was identified as an independent risk factor for SSI. This finding is supported by study done at [22, 31, 43]. The possible explanation might be prolonged surgery increases the exposure of tissues to the environment, raising the risk of contamination by microorganisms. Additionally, extended operative time is often associated with more complex procedures, greater tissue manipulation, and prolonged use of invasive devices, all of which compromise wound healing and increase susceptibility to infection. And also extended surgical time may also lead to greater blood loss and reduced immunity, further exacerbating the likelihood of SSI.

Women with a post‐operative hematocrit level of less than 30% had 4.3 times higher odds of developing surgical site infections compared to those with hematocrit levels of 30% or more. This finding supported study conducted in various settings in Ethiopia [17, 23, 30, 42, 43, 45]. The Possible Explanation might be Low hematocrit levels (< 30%) indicate anemia, which compromises oxygen delivery to tissues and impairs wound healing. Suboptimal oxygenation and decreased immune function can delay the resolution of bacterial contamination at the surgical site, increasing the risk of infection.

Another factor significantly associated with SSI after cesarean delivery was the lack of vaginal preparation prior to the procedure. Mothers who did not undergo vaginal preparation were 4 times more likely to develop SSI compared to those who received vaginal preparation. This finding is supported by studies conducted in [46]. The possible explanation might be lack of vaginal preparation increases the risk of introducing pathogenic microorganisms from the vaginal flora to the surgical site during the procedure. Vaginal preparation, typically involving antiseptic cleansing, significantly reduces bacterial load and minimizes contamination during cesarean delivery. The absence of this preventive measure may result in an elevated risk of SSI, particularly in environments where bacterial colonization is high or procedures are prolonged. This finding underscores the importance of incorporating routine preoperative vaginal preparation into infection prevention protocols to enhance surgical outcomes.

The clinical implication of this study is the potential to improve maternal care through enhanced infection control practices, optimized perioperative management, and tailored interventions to address modifiable risk factors, thereby reducing morbidity and improving surgical outcomes. The scientific contribution of this study is its identification of key risk factors, such as chorioamnionitis, prolonged surgical duration, and intraoperative blood loss, which provide an evidence base for developing targeted preventive strategies, particularly in resource‐limited settings.

### 4.1 Strength of the Study and Limitations of the Study

The study's strength lies in its inclusion of understudied variables, such as the absence of vaginal preparation, which provides novel insights into associated factors for surgical site infections. This adds valuable evidence to existing knowledge, particularly in resource‐limited settings.

This study employs a retrospective record review, which inherently limits the availability and completeness of data. Missing or unregistered information on essential socio‐demographic and clinical characteristics may introduce bias and affect the robustness of the findings. Additionally, reliance on existing records restricts the ability to explore unrecorded variables or verify data accuracy, potentially impacting the depth of analysis.

## 5 Conclusion

This study found that the magnitude of post‐cesarean surgical site infection at WSUCSH was 8.8%, representing a clinically significant burden consistent with rates reported across Ethiopian hospitals. Prolonged operative duration, prolonged rupture of membranes, chorioamnionitis, inadequate vaginal antiseptic preparation, excessive intraoperative blood loss, and postoperative hematocrit below 30% were identified as independent predictors of SSI. Notably, all significant risk factors identified are modifiable, suggesting that a substantial proportion of post‐cesarean SSIs in this setting are preventable through targeted perioperative interventions.

Strengthening adherence to evidence‐based infection prevention practices particularly vaginal antiseptic preparation, timely management of obstetric complications, and correction of maternal anemia is essential to reducing SSI‐related maternal morbidity in resource‐limited settings. These findings provide locally relevant, actionable evidence to guide clinical practice, institutional policy, and future research aimed at improving maternal surgical outcomes in Southern Ethiopia.

## 6 Recommendations

Given that prolonged operative duration was a significant predictor of SSI, surgical teams should adopt structured operative checklists and ensure adequate preoperative preparation to minimize unnecessary delays during cesarean delivery. Since prolonged rupture of membranes substantially increased infection risk, obstetric units should establish clear triage protocols for early identification and timely surgical intervention in women presenting with prolonged membrane rupture, avoiding unnecessary delays in decision‐to‐incision time. The strong association between chorioamnionitis and SSI underscores the need for prompt clinical diagnosis and initiation of broad‐spectrum antibiotics prior to surgery, and infection screening should be integrated into routine preoperative assessment. As inadequate vaginal antiseptic preparation was among the strongest predictors identified, hospitals should institutionalize preoperative vaginal preparation with chlorhexidine or povidone‐iodine as a non‐negotiable standard of care for all cesarean deliveries, supported by regular staff training and supply availability. To address excessive intraoperative blood loss, surgical teams should follow meticulous hemostatic techniques, maintain availability of blood products, and have transfusion protocols readily accessible in the operating theater. Finally, since postoperative hematocrit below 30% independently predicted SSI, routine postoperative hematocrit monitoring should be implemented for all cesarean delivery patients, with timely correction of anemia through iron supplementation or transfusion to support immune function and wound healing during recovery.

## Author Contributions

**Kidane Tsegaw:** conceptualization, investigation, methodology, writing – original draft, software, formal analysis, project administration. **Tesfaw Alemu Legas:** writing – original draft, methodology, software, formal analysis, data curation. **Amene Abebe Kerbo:** validation, visualization, supervision, data curation, methodology. **Belachew Shote Garbo:** visualization, validation, data curation, supervision, methodology. **Samuel Minalkew:** methodology, formal analysis, software, data curation, validation. **Tigist Nega Alemu:** writing – review and editing, methodology, validation, visualization, data curation. **Wondimagegn Genaneh Shiferaw:** methodology, validation, visualization, writing – review and editing, data curation.

## Funding

This research did not receive any specific grant from funding agencies in the public, commercial, or not‐for‐profit sectors, and consequently no funding body had any role in the study design; collection, analysis, or interpretation of data; writing of the report; or the decision to submit the article for publication.

## Ethics Statement

The study was conducted in accordance with local regulations, institutional requirements, and the principles of the Declaration of Helsinki [47]. Ethical approval for this study was obtained from the Ethical Review Committee of Wolaita Sodo University, College of Health Sciences and Medicine, with approval number CHSM/ERC/02/15. As the study employed a retrospective chart review design with no direct patient contact, informed consent was waived by the IRB. Patient confidentiality was strictly maintained, and all data were anonymized prior to analysis.

## Consent

Ethical approval was obtained from the Ethical Review Committee of Wolaita Sodo University, College of Health Science and Medicine. This study was a retrospective chart review, and informed consent was waived by the Wolaita Sodo University, College of Health Science and Medicine, Ethical review Committee.

## Conflicts of Interest

The authors declare no conflicts of interest.

## Transparency Statement

Dr. Kidane Tsegaw (MD), as the article guarantor, affirms that this article is an honest, accurate, and transparent account of the study being reported; that no important aspects of the study have been omitted; and that any discrepancies from the study as planned have been explained.

## Data Availability

This study was based on data extracted directly from patient medical charts at Wolaita Sodo University Comprehensive Specialized Hospital. The derived data supporting the findings of this study are not publicly available due to patient confidentiality constraints but are available from the Principal Investigator, Dr. Kidane Tsegaw (MD), Department of Medicine, Wolaita Sodo University School of Medicine (email: kidanetsegaw@gmail.com, upon reasonable request.
